# Case Report: Long-term metabolic response of metastatic uveal melanoma to pembrolizumab on FDG-PET/CT despite a serial pseudoprogressions phenomenon

**DOI:** 10.3389/fimmu.2023.1243208

**Published:** 2023-12-04

**Authors:** Karim Amrane, Coline Le Meur, Philippe Thuillier, Jacques Dzuko Kamga, Pierre Alemany, Frederic Chauvelot, Clémence Niel, Alex Bellange, Ronan Abgral

**Affiliations:** ^1^ Department of Oncology, Centre Hospitalier des Pays de Morlaix, Morlaix, France; ^2^ Inserm, UMR1227, Lymphocytes B et Autoimmunité, Univ Brest, Inserm, LabEx Immunotherapy-Graft-Oncology (IGO), Brest, France; ^3^ Department of Radiotherapy, University Hospital of Brest, Brest, France; ^4^ Department of Endocrinology, University Hospital of Brest, Brest, France; ^5^ Department of Nuclear Medicine, University Hospital of Brest, Brest, France; ^6^ Department of Pathology, Ouestpathology Brest, Brest, France; ^7^ Department of Onco-pharmacy, Centre Hospitalier des Pays de Morlaix, Morlaix, France; ^8^ Unité Mixte de Recherche (UMR) Inserm 1304 Groupe d'étude de la thrombose de Bretagne-Occidentale (GETBO), Institut Federatif de Recherche (IFR) 148, University of Western Brittany, Brest, France

**Keywords:** uveal melanoma, pembrolizumab, FDG-PET, immune checkpoint inhibition, pseudoprogression

## Abstract

Uveal melanoma (UV) is a rare and aggressive melanoma with poor 1-year survival. up to 50% of UV patients develop metastases, mainly to the liver. Here, the authors present a 2-deoxy-2-[^18^F] fluoro-D-glucose positron emission tomography (^18^F-FDG-PET) study of a very rare case of secondarily metastatic UV in an 81-year-old Caucasian with a dramatic response to pembrolizumab associated with serial pseudogression. ^18^F-FDG-PET associated with clinical status and peripheral blood derived neutrophil-to-lymphocyte ratio (dNLR) were performed to guide therapeutic strategy due to an atypical pseudoprogression phenomenon.

## Introduction

Uveal melanoma (UV) accounts for less than 5% of all melanomas (1). Although UV arises from uveal melanocytes, it differs from cutaneous melanoma (CM) in its oncogenic drivers, development, and tumor microenvironment, with different molecular drivers and a cold tumor microenvironment compared to CM (2, 3). These differences likely contribute to a poor clinical response to systemic therapy, including immune checkpoint inhibition (ICI), which rarely induces durable remissions in patients with metastatic UV (4–6). Up to 50% of patients with UV develop metastases, primarily to the liver (7, 8), which affects prognosis with a median overall survival of approximately 1 year (9).

## Case description

We present the case of an 81-year-old Caucasian man with a previous medical history of non-mutated right eye UV (BRAF/NRAS/c-Kit wild-type), treated by surgery 16 years ago and in complete remission since then. He was referred to our oncology department because of a histologically proven unresectable liver recurrence, which was detected by ultrasound as part of the surveillance performed since surgery.

A 2-deoxy-2-[^18^F] fluoro-D-glucose positron emission tomography (FDG-PET/CT) scan was performed for stating and showed pathological hypermetabolism (SUVmax 5.7) in a large isolated right hepatic hypodense area with no other lesions, particularly locoregional lymph nodes. A complementary magnetic resonance imaging (MRI) scan of the brain showed no metastasis. A pre-therapeutic work-up including lactate dehydrogenase (LDH) assay (assessed as 165 u/l, i.e. within the norm) (10–12) and peripheral blood derived neutrophil-to-lymphocyte ratio (dNLR) was calculated with a favorable score of 2.15 (<3) (13), classifying the disease as having a favorable immune prognostic index (IPI) (14). Anti PD-1 immune checkpoint inhibitor (ICI) therapy with pembrolizumab every 3 weeks was initiated (15).

**Figure 1:**
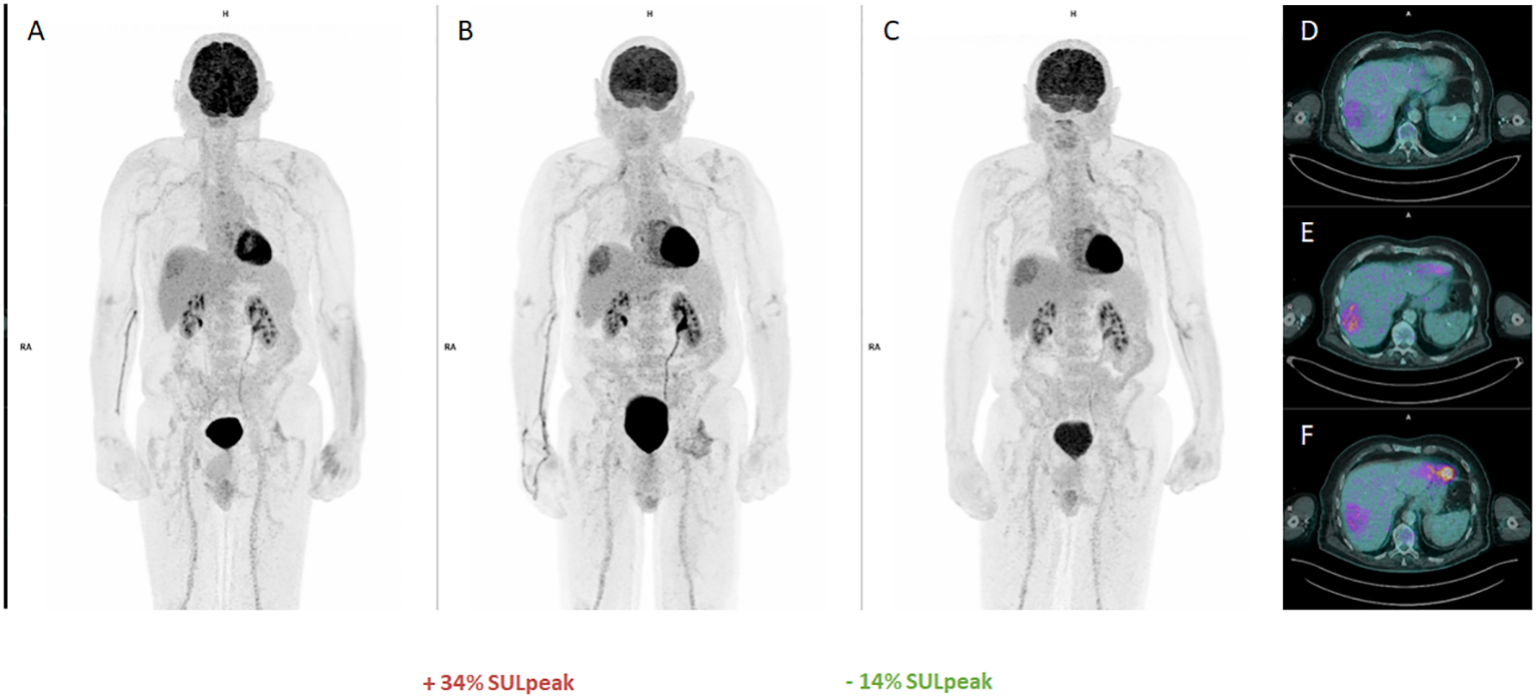
FDG-PET first pseudoprogression : (**A, D**: MIP and Axial baseline), (**B, E** : MIP and Axial unconfirmed progressive metabolic disease (uPMD) with 28% increase in SULpeak of the main lesion, (**C, F**: MIP and Axial confirming a pseudoprogression (PsPD).

After 3 cycles of pembrolizumab, FDG-PET/CT (**Figure 1B** FDG-PET MIP, **Figure 1E** Axial FDG-PET/CT: assessment after cycle 3 of pembrolizumab) was in favor of unconfirmed progressive metabolic disease (uPMD) according to iPERCIST criteria (16, 17) with an increase of 34% in lean body mass corrected SUV peak (SULpeak) (>30%) compared to baseline (**Figure 1A** FDG-PET MIP, **Figure 1D** Axial FDG-PET/CT: initial staging), without new hypermetabolism. After 2 new cycles of pembrolizumab, a close evaluation was performed in order not to confirm or not a proven progression. The result showed a decrease in SULpeak of more than 15%, confirming a pseudoprogression (PsPD) (**Figure 1C** FDG-PET MIP, **Figure 1F** Axial FDG-PET/CT: assessement after cycle 5 of pembrolizumab). In parallel with this episode of PsPD, the patient developed rheumatoid arthritis grade 2 according to the Common Terminology Criteria for Adverse Events (CTCAE - version 5.0) classification (18), which resolved rapidly with short-term corticosteroid therapy.

**Figure 2:**
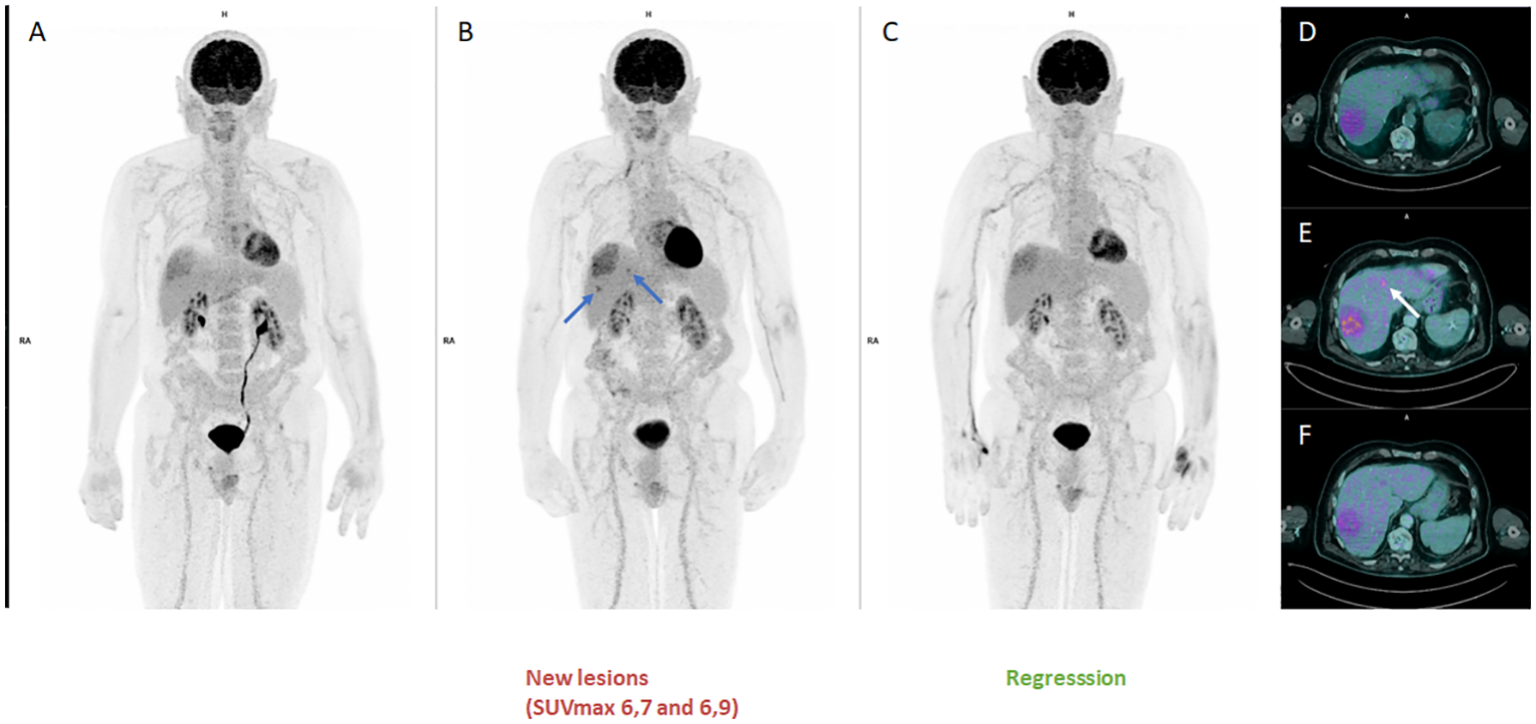
FDG-PET second pseudoprogression : (**A, D**: MIP and Axial disease stable before progression), (**B, E** : MIP and Axial disease progression according to PERCIST criteria with 28% increase in SULpeak of the main lesion and the appearance of 2 new lesions), (**C, F**: MIP and Axial confirming a pseudoprogression (PsPD) with 15% decrease in SULpeak with the disappearance of the 2 new hypermetabolisms corresponding to a uPMD according to iPERCIST criteria.

The disease remained stable for more than 1 year with excellent clinical tolerability (**Figure 2A** FDG-PET MIP, **Figure 2D** Axial FDG-PET/CT). After 21 cycles of pembrolizumab, a PET scan showed a 28% increase in SULpeak of the main lesion and the appearance of 2 new lesions (**Figure 2B** FDG-PET MIP, **Figure 2E** Axial FDG-PET/CT, arrows SUVmax 6,7 and 6,9), consistent with progression according to PERCIST criteria (19). Given the good general condition and excellent tolerability of the patient, it was decided to continue treatment until cycle 23. At the same time, a liver biopsy was performed, which revealed the presence of disease without specific lymphocytic infiltrate. The early re-assessment (**Figure 2C** FDG-PET MIP, **Figure 2F** Axial FDG PET-CT) showed a further 15% decrease in SULpeak with the disappearance of the 2 new hypermetabolisms seen on the previous scan thus corresponding to a uPMD according to iPERCIST criteria (16, 17).

**Figure 3:**
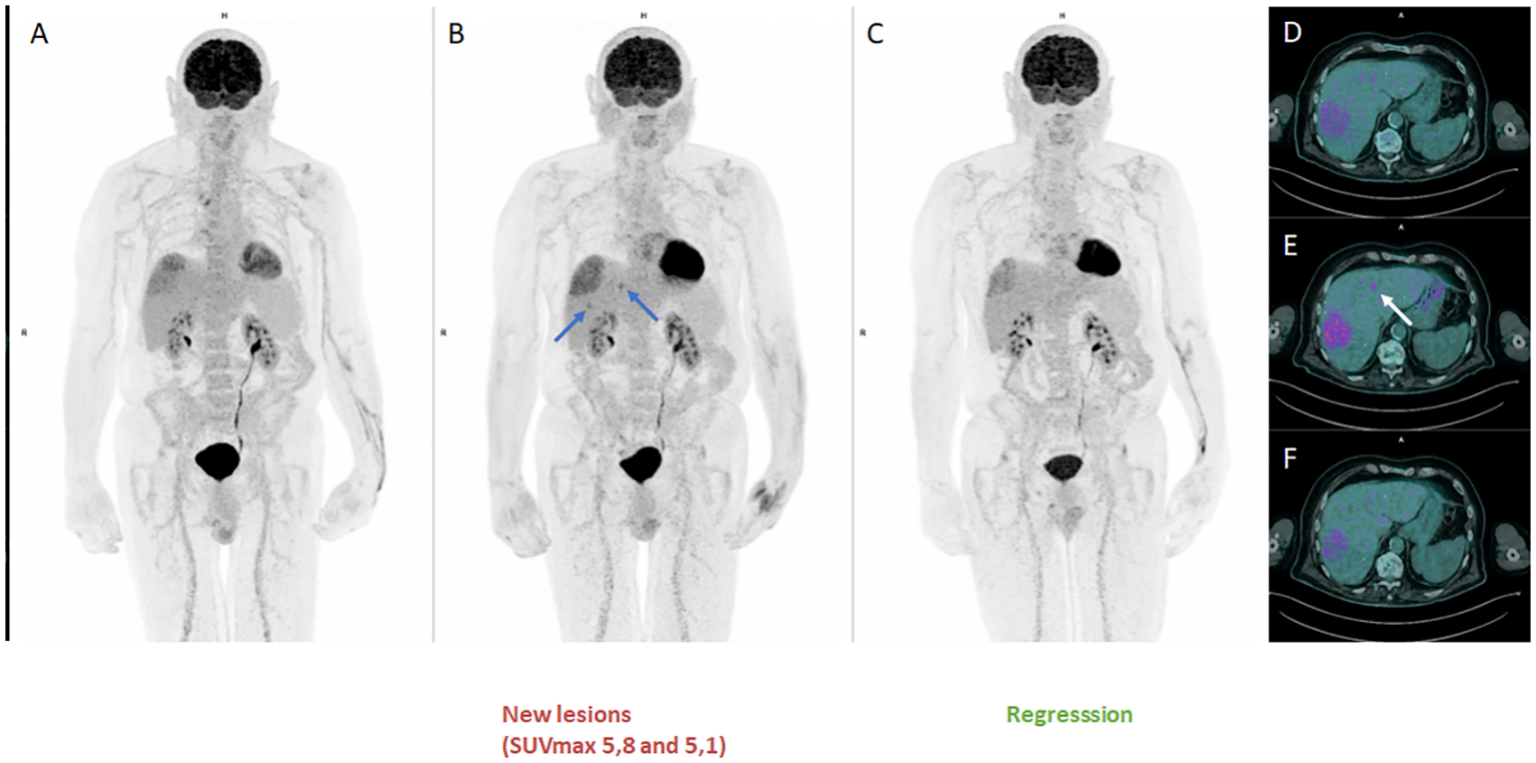
FDG-PET MIP third pseudoprogression (**A, D**: MIP and Axial disease stable before progression), (**B, E** : MIP and Axial disease progression according to PERCIST criteria with 27% increase in SULpeak of the main lesion and the recurrence of the 2 previous lesions), (**C, F**: MIP and Axial confirming a pseudoprogression (PsPD) with a stable SULpeak with renewed disappearance of the 2 hypermetabolisms seen on the previous scan.

The disease remained stable for more than 3 months with excellent clinical tolerability (**Figure 3A** FDG-PET MIP, **Figure 3D** Axial FDG-PET/CT). After 34 cycles of pembrolizumab, a PET scan showed a 27% increase in SULpeak of the main lesion and the recurrence of the 2 previous lesions (**Figure 3B** FDG-PET MIP, **Figure 3E** Axial FDG-PET/CT, arrows SUVmax 5,1 and 5,8) described in **Figure 2**, consistent with progression (19). Once again, it was decided to continue treatment until cycle 38. The early re-assessment showed a stable SULpeak with renewed disappearance of the 2 hypermetabolisms seen on the previous scan (**Figure 3C** FDG-PET MIP, **Figure 3F** Axial FDG-PET/CT).

Finally, 3 months after cycle 38 of ICI treatment, the patient presented confirmed disease progression, with an unequivocal metabolic increase in the main lesion of the hepatic dome and the appearance of multiple other FDG-avid liver metastasis. A new line of treatment was proposed with tebentafusp (20). Disease control with pembrolizumab lasted a total of 28months in this patient.

## Discussion

This case showed a dramatic response to anti PD1 (28 months) but also a series of PsPD (3 times), which is exceptional because it is the first reported in UV and the second reported in melanoma (21). However, the first published report concerned a 2 times PsPD in a metastatic (liver and bone) CM, treated with nivolumab. Indeed, they found a 118% increase in the size of liver lesion after 3 months starting nivolumab followed by regression; then the appearance of a peritoneal nodule at 8 months, which decreased at 12 months.

ICI immunotherapy has revolutionized cancer management, but it soon became apparent that a subset of patients treated with ICI had an atypical tumor response profile, either after an increase in tumor burden or after the appearance of new lesions, a phenomenon known as pseudoprogression disease (PsPD), which is classified as progressive disease by conventional response criteria (RECIST in CT and PERCIST in PET) (22). Several teams have developed different criteria to take into account this specificity associated with the immune response, such as in conventional imaging the immune-related response criteria (irRC) (23), the immune-related solid tumor response evaluation criteria (irRECIST) (24, 25) and iRECIST (26); and in PET imaging the iPERCIST and imPERCIST criteria (16, 17, 27).

Park et al. reported in a meta-analysis that the incidence of PsPD in clinical trials assessing immunotherapy was 6.0%. PsPD is defined as progressive disease followed by stable disease or partial or complete response (28). The incidence of PsPD by tumor type was 6.4% for melanoma, 5.0% for non-small cell lung cancer and 7.0% for genitourinary cancer. The incidence of PsPD with PD-1/PD-L1 inhibitors alone was 5.7% (95% CI: 4.8%, 6.6%), while it was estimated at 9.7% with anti-CTLA-4 (28).

In an analysis of 32 patients with a variety of tumors, Monch et al. studied the characteristics of PsPD, relatively concordant with our case report. They found that PsPD occurs in 81% of cases after the first treatment response assessment as immune unconfirmed progressive disease (iUPD), with a tumor burden growth regularly below +100%. PsPD was associated with a significant increase in progression of both targeted and non-targeted lesions. LDH levels in PsPD patients were normal in most cases and 40% of patients with PsPD had adverse events (29).

The CD3+, CD4+, CD8+, TIA1+ and granzyme B+ lymphoid infiltrate found in the tumor biopsy could be a strong argument in favor of PsPD (30, 31). In our case, we did not perform a biopsy at the time of the first PsPD because we believed that this phenomenon was well known in literature and that an early re-assessment could spare the patient an invasive procedure. Nevertheless, a biopsy was performed at the time of the second PsPD episode, a rare situation as above mentioned. But it did not show a particularly large lymphoid infiltrate, which could be explained by the fact that the specimen was not infiltrated by lymphocytes.

**Figure 4:**
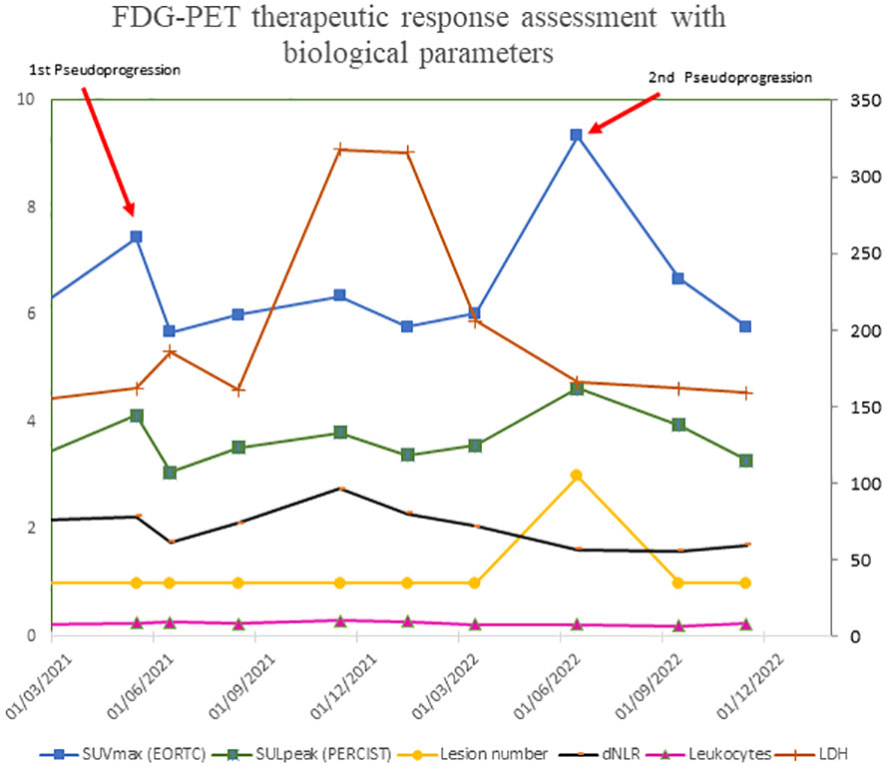
FDG-PET therapeutic response assessment with biological parameters.

In contrast, we had performed biological monitoring based on the number of leukocytes and neutrophils in peripheral blood during treatment to calculate dNLR (32) and LDH (10–12). As shown in **Figure 4**, normal LDH levels, dNLR <3 and IPI score were associated with maintenance of good overall clinical status throughout treatment, suggesting ongoing activation of antitumor immune responses that may lead to this phenomenon of serial PsPD. However, this biological monitoring does not appear to be as robust as measuring circulating ctDNA to distinguish PsPD from proven progression. Indeed, Lee et al. demonstrate that ctDNA profiles can accurately differentiate PsPD from true disease progression in melanoma patients treated with anti PD-1 therapy, with a sensitivity of 90% (95% CI, 68%-99%) and a specificity of 100% (95% CI, 60%-100%) for predicting PsPD (33).

Finally, the choice of interpretation criteria in the context of ICI immunotherapy is critical. Although the RECIST V.1.1 criteria are the gold standard for assessing treatment response, progression or stable disease in patients with solid tumors following cancer therapy (22), they do not take into account unconventional response patterns, such as PsPD in ICI therapy.

To date, the two main RECIST1.1-derived and immunotherapy-adapted assessment criteria used in clinical practice for morphological imaging are irRECIST and iRECIST. Nishino et al. proposed the irRECIST criteria, a system based on unidimensional assessment and a lower number of target lesions than the RECIST 1.1 criteria (24). The main difference between irRECIST and RECIST 1.1 is how new lesions are included in response assessment. In contrast to RECIST 1.1, where new lesions are immediately equated with PD, irRECIST includes new lesions in the total measured tumor burden (TMTB). This method ensures that potentially effective treatment is not interrupted when new lesions appear (34). In addition, a comparison of the 2 criteria for evaluating immunotherapy showed a discrepancy of 8.3% (35). Confirmation of progression is recommended for patients with a minimal increase in TMTB of more than 20%, particularly during the first 12 weeks of treatment, in order to distinguish PsPD from progression (24, 25).

The iRECIST criteria are similar to RECIST 1.1 and irRECIST in terms of recommended imaging modalities, definitions of measurable lesions and target lesions. However, target and non-target lesions are not counted together. Therefore, unlike irRECIST, they are not added to the largest dimension of all target lesions (26). Once iUPD has been identified, re-assessment should be carried out 4 to 8 weeks later to allow for continuity of treatment, but also for salvage therapy, if necessary (34).

In this case, we used FDG-PET/CT to assess treatment response, as recommended by French and European guidelines (36, 37). As with functional imaging, PERCIST-based criteria adapted to immunotherapy have been developed, the most recent being iPERCIST and imPERCIST (16, 27). It has been shown that while imPERCIST reduces the overdiagnosis of progressive disease, new lesions in patients with partial metabolic response or stable metabolic disease were ultimately found to be metastases in 55% of cases. Thus, the prognosis for patients whose target lesions are shrinking or stable but who develop new lesions appears indeterminate. Therefore, histological confirmation by biopsy should be considered before changing treatment (27).

This case highlights the usefulness of a combined clinical-biological and FDG-PET/CT approach in assessing response to ICI and also suggests that the appearance of a new hypermetabolic lesion should not routinely be equated with ICI treatment failure.

## Data availability statement

The original contributions presented in the study are included in the article/supplementary material. Further inquiries can be directed to the corresponding author.

## Ethics statement

Written informed consent was obtained from the individual(s) for the publication of any potentially identifiable images or data included in this article.

## Author contributions

KA and RA drafted an initial version of the manuscript and are the guarantors of the case report. KA, CL, FC, CN, and AB provided the patient details. PT, JD, and RA provided image analysis. PA provided histological analysis. All authors contributed to the article and approved the version submitted for publication.
